# Neonatal regulatory T cells persist into adulthood across multiple tissues with high enrichment in the skin

**DOI:** 10.1126/sciadv.adx8037

**Published:** 2025-10-03

**Authors:** Morgane Hilaire, Angélina Mimoun, Léonie Cagnet, Rémy Villette, Aristeidis Roubanis, Hugo Sentenac, Benoît L. Salomon

**Affiliations:** ^1^ Sorbonne Université, INSERM U1135, CNRS, Centre d’Immunologie et des Maladies Infectieuses (CIMI-Paris), F-75013, Paris, France.; ^2^ Toulouse Institute for Infectious and Inflammatory Diseases (Infinity), INSERM UMR1291–CNRS UMR5051–University Toulouse III, Toulouse, France.

## Abstract

Foxp3^+^ regulatory T cells (T_regs_) reside in both lymphoid and nonlymphoid organs, where they play a crucial role in immune tolerance and tissue homeostasis. In mice, T_regs_ begin colonizing these tissues shortly after birth, contributing to long-term immune response regulation therein. However, the kinetics of T_reg_ generation across different tissues remains unclear. Here, we investigate T_reg_ ontogeny from birth to adulthood in various tissues. In lymphoid organs, the adult T_reg_ pool is continuously replenished with cells generated at different ages. In contrast, the skin retains a large fraction of T_regs_ that colonize the tissue during the neonatal period, with minimal turnover in adulthood. The liver, lungs, and colon exhibit intermediate T_reg_ renewal dynamics. Notably, neonatal T_regs_ that persist into adulthood display a more activated phenotype and express markers associated with tissue-resident T_regs_ and type 2 immunity. Our findings reveal tissue-specific differences in T_reg_ generation kinetics and highlight a major phenotypic shift between neonatal and adult-derived T_regs_.

## Teaser

The adult skin T_reg_ pool is predominantly of neonatal origin, in contrast to other tissues with higher renewal throughout life.

## INTRODUCTION

Mice are born lymphopenic, and T cells begin to emerge from the thymus and progressively colonize peripheral tissues only after birth. Mice are thus an ideal model for studying T cell ontogeny and the progressive establishment of immune homeostasis throughout the first few weeks of life. During this critical window, the immune system must rapidly establish effective defenses against pathogens while simultaneously promoting tolerance to self-antigens, the microbiota, and food antigens.

Neonatal and adult T cell immunity differs quite remarkably (*1*). Hallmark features of neonates include lymphopenia-induced proliferation, a T helper cell 2 (T_H_2)–skewed bias, and a T cell receptor repertoire that is both restricted and broadly cross-reactive. This period is also highly permissive for the generation of Foxp3^+^ regulatory T cells (T_regs_), which arise from both thymic and peripheral origins (*2*, *3*). Studies have explored the kinetics of T_reg_ colonization in various tissues, revealing distinct spatial and temporal patterns. In secondary lymphoid organs such as the spleen and lymph nodes (LNs), T_regs_ of neonates are present in low proportions (*4*, *5*). Higher T_reg_ proportions are observed in the liver and lungs, where they peak at 15 to 30% of CD4^+^ T cells 2 weeks after birth (*4*–*6*). In the liver, early T_reg_ colonization limits local activation of conventional T cells (Tconvs) (*6*). Notably, the skin undergoes a particularly high level of T_reg_ accumulation, with these cells constituting up to 80% of CD4^+^ T cells by 1 to 2 weeks of age, followed by a gradual decline (*5*, *7*, *8*).

A subset of T_regs_ generated in neonates (neonatal T_regs_) persists in the adult spleen, where they exhibit higher activation, superior suppressive capacity, and an autoreactive-biased repertoire compared to those generated in adulthood (*9*). Neonatal T_regs_ play an essential role in preventing autoimmunity in adulthood (*9*, *10*). In the skin, neonatal T_regs_ establish long-term tolerance to commensal microbes and dampen local T_H_2 responses in adults (*7*, *11*). However, whether their sustained immunoregulatory effects are due to their prolonged persistence or the induction of infectious tolerance remains unknown.

Despite their critical role in immune homeostasis, the precise ontogeny and generation kinetics of the adult T_reg_ pool across tissues remains undetermined. While it has been shown that neonatal T_regs_ persist in the spleen, their proportion in the adult T_reg_ compartment remains poorly quantified (*9*). Here, we addressed these questions by exploiting a fate-mapping system to track T_reg_ generation across different developmental stages and tissues. We demonstrate that, in lymphoid organs, the adult T_reg_ pool is continuously replenished throughout life. In contrast, most T_regs_ in adult skin originate from cells generated during the neonatal period, with early-life colonization shaping the adult T_reg_ pool. Furthermore, neonatal T_regs_ exhibit a distinct phenotypic signature, characterized by elevated expression of activation markers and molecules associated with tissue residency and type 2 immunity.

## RESULTS

### Experimental approach

**Fig. 1.**
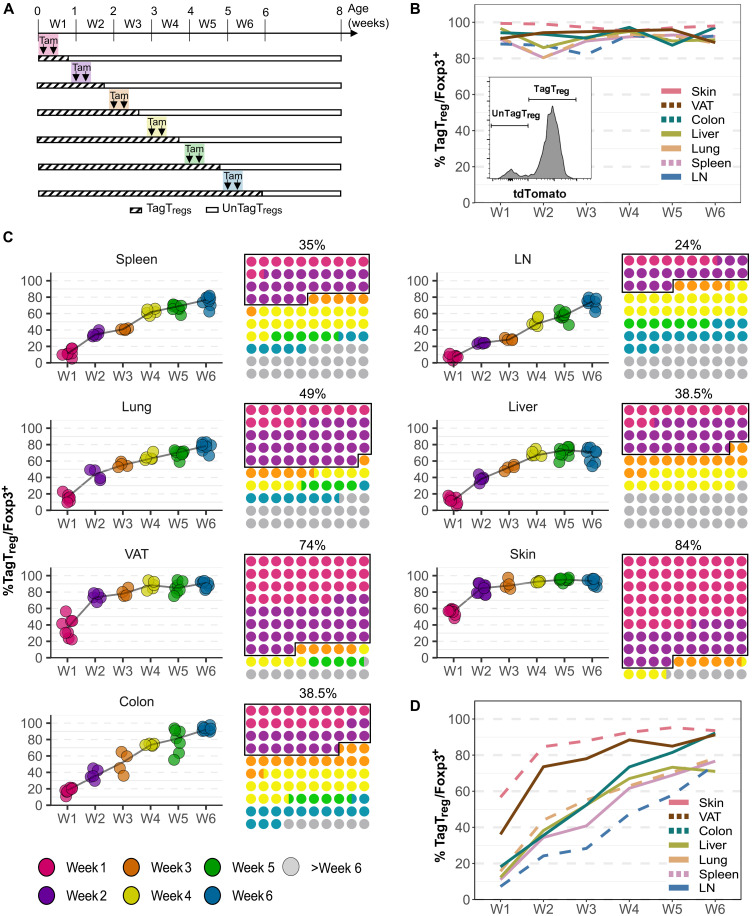
Dynamics of the generation of the T_reg_ pool in 8-week-old mice. *Foxp3^iCre^ R26^Tom^* mice were treated with tamoxifen either at week (W) 1 or 2 or 3 or 4 or 5 or 6 of life and were analyzed by flow cytometry at 8 weeks of age (A, C, and D) or 7 days after the beginning of tamoxifen treatment (B). (**A**) Experimental design showing the different mouse groups. (**B**) Proportion of TagT_regs_ among Foxp3^+^ cells at day 7 posttreatment from the spleen, LNs, lungs, liver, colon, and skin. The histogram represents the gating strategy used to discriminate unTagT_regs_ (Foxp3^+^ tdTomato^−^) and TagT_regs_ (Foxp3^+^ tdTomato^+^). (**C**) Proportion of TagT_regs_ among Foxp3^+^ cells in the indicated tissues in 8-week-old mice treated with tamoxifen at weeks 1 to 6 (graphs, each symbol represents one mouse). These values enabled the calculation of the proportion of T_regs_ generated at week 1 to week 6 among the T_reg_ pool of 8-week-old mice (10 × 10 dot plots). The pool of T_regs_ generated before 2 weeks of life was outlined, and their proportion was indicated. (**D**) To highlight differences between organs, the different graph curves (without symbols) shown in (C) were superimposed on one another in a single graph. Data were from multiple independent experiments with four mice per group (weeks 2, 3, and 4) or eight mice per group (weeks 1, 5, and 6).

To investigate T_reg_ ontogeny, we used a fate-mapping strategy by crossing *Foxp3^tm9(EGFP/cre/ERT2)Ayr^*/J (*Foxp3^iCre^*) mice with B6.Cg-Gt(ROSA)26Sor^tm9(CAG-tdTomato)Hze/J^ (*R26^Tom^*) mice. In this system, the *Foxp3^iCre^* construct ensures T_reg_-specific expression of the *Cre-ERT2* recombinase, which remains cytoplasmic until tamoxifen administration triggers its nuclear translocation. The *R26^Tom^* allele contains a loxP-flanked STOP cassette upstream of the *tdTomato* reporter. Upon tamoxifen treatment, Cre-mediated recombination excises the STOP cassette, leading to stable tdTomato expression in existing T_regs_, termed TagT_regs_. T_regs_ arising posttreatment, which lack tdTomato expression, are designated as unTagT_regs_. To determine the generation kinetics of the adult T_reg_ pool, we administered tamoxifen at various ages from birth to 6 weeks. All mice were analyzed at 8 weeks across multiple tissues, including the spleen, LNs, lungs, liver, visceral adipose tissue (VAT), colon, and skin (Fig. 1A).

To validate this approach, we confirmed specificity of tdTomato labeling to T_regs_ upon tamoxifen induction (fig. S1A). In addition, we assessed the efficiency of tdTomato T_reg_-tagging across tissues by analyzing mice 7 days post–tamoxifen administration, which represents the optimal timing (fig. S1B). The proportion of TagT_regs_ was consistently high, more than 90% in most conditions and nearly 100% in the skin (Fig. 1B). Last, we evaluated the duration of tamoxifen activity by transferring T_regs_ from *Foxp3^iCre^ R26^Tom^* mice into T_reg_-depleted recipients (fig. S1C). Analysis revealed peak recombination activity 1 to 5 days post–tamoxifen injection, with no more activity after 8 days (fig. S1D). Thus, our system robustly labels T_regs_ while ensuring transient tamoxifen exposure.

### Tissue-specific kinetics of T_reg_ pool generation

To determine the temporal dynamics of T_reg_ generation, *Foxp3^iCre^ R26^Tom^* mice were treated with tamoxifen during the first to the sixth weeks of life, and T_regs_ were analyzed at 8 weeks across seven tissues (Fig. 1A). This protocol enabled retrospective investigation of the ontogeny of T_regs_ from birth to 8 weeks of age. Tamoxifen treatment during the first week of life marked T_regs_ generated at week 1. Treatment during the second week of life marked T_regs_ generated at weeks 1 and 2. By difference, we could then calculate the proportion of T_regs_ generated at week 2. Similarly, treatments carried out the third, fourth, fifth, and sixth weeks of life identified T_regs_ generated in weeks 3, 4, 5, and 6, respectively (Fig. 1A). We represented the composition of the T_reg_ pool of 8-week-old mice in two ways: with graphs showing the progressive accumulation of newly generated T_regs_ (Fig. 1C, left) and with 10 × 10 dot plots depicting the composition of the T_reg_ pool according to their generation stage (Fig 1C, right). In lymphoid tissues (spleen and LNs), newly generated T_regs_ accumulated gradually. Approximately 25% of LN T_regs_ and 35% of spleen T_regs_ were generated by week 2, rising to 50 and >60% by week 4, respectively (Fig. 1C). Nonlymphoid tissues exhibited distinct kinetics. In the lungs, T_regs_ accumulated rapidly, with 50% generated by week 2. In liver and colon, T_reg_ generation increased sharply, with ~70% TagT_regs_ by week 4. T_regs_ in VAT and skin exhibited the most pronounced neonatal bias; 75% of VAT T_regs_ and 85% of skin T_regs_ were generated by week 2, with 60% of skin T_regs_ arising within the first week of life (Fig. 1C). The comparison of T_reg_ pool generation kinetics between tissues is highlighted in Fig. 1D. Collectively, these findings highlight tissue-specific differences in T_reg_ ontogeny: Lymphoid tissues maintain continuous T_reg_ renewal, whereas VAT and skin predominantly establish their T_reg_ pool during early life.

To determine whether T_reg_ ontogeny correlates with tissue colonization, we analyzed the proportion of T_regs_ among CD4^+^ T cells across ages (fig. S2). In lymphoid tissues, T_reg_ proportions remained relatively stable (10 to 18%) between 1.5 and 6.5 weeks of age. In the liver, an early peak (30% at 1.5 weeks) was followed by stabilization at 15 to 18%. Lungs exhibited a similar pattern but with a milder early peak. In skin, T_regs_ constituted 70 to 80% of CD4^+^ T cells between 1.5 to 3.5 weeks of age, dropping to 20 to 30% after 4.5 weeks. This kinetics align with our T_reg_ ontogeny data, suggesting that kinetics of tissue T_reg_ colonization dictates cell composition.

### Neonatal T_regs_ persisting in adults preferentially express GATA3

**Fig. 2.**
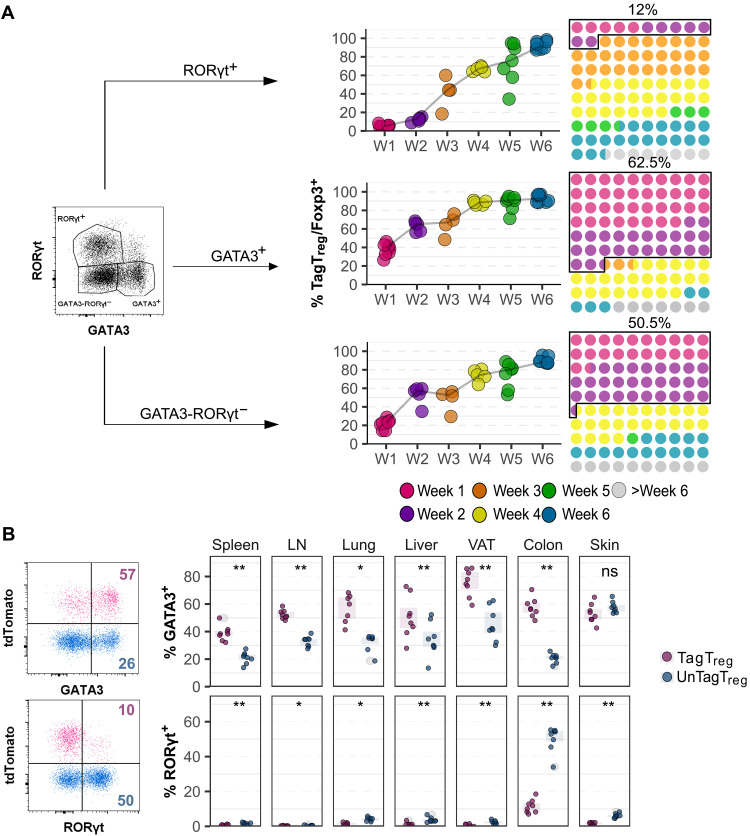
Neonatal T_regs_ expressed more GATA3. (**A**) *Foxp3^iCre^ R26^Tom^* mice were treated with tamoxifen at weeks 1 to 6 and were all analyzed at 8 weeks of age as in Fig. 1. Proportion of TagT_regs_ among GATA3^+^, RORγt^+^, and GATA3^−^RORγt^−^ T_regs_ in the colon (graphs, each symbol represents one mouse). These values enabled the calculation of the proportion of T_regs_ generated at week 1 to week 6 among the three subtypes of colonic T_regs_ of 8-week-old mice (10 × 10 dot plots). The pool of T_regs_ generated before 2 weeks of life was outlined, and their proportion was indicated. (**B**) *Foxp3^iCre^ R26^Tom^* mice were treated with tamoxifen at week 1 and analyzed at 8 weeks of age to compare the proportion of GATA3^+^ and RORγt^+^ among unTag and TagT_regs_ in the seven analyzed tissues. Representative dot plots from the colon are shown. Data were from multiple independent experiments with four mice per group (weeks 2, 3, and 4) or eight mice per group (weeks 1, 5, and 6) (B) mice per group. Statistical significance was calculated using a nonparametric Wilcoxon test. Each symbol represents one mouse, and the bars represent the medians. *P < 0.05 and **P < 0.01. ns, not significant.

T_regs_ in the colon comprise Helios/GATA3^+^, RORγt^+^, and double-negative (GATA3^−^RORγt^−^) subsets (*12*). Neonatal T_regs_ preferentially differentiate into GATA3^+^ T_regs_, while adult-derived T_regs_ exhibit a bias toward RORγt^+^ differentiation (*13*). Using our fate-mapping approach, we assessed whether neonatal T_regs_ would persist into adulthood and retain their identity. At 8 weeks, more than 60% of GATA3^+^ T_regs_ were generated before 2 weeks of age, whereas only 12% of RORγt^+^ T_regs_ originated from this early life period (Fig. 2A). The development kinetics of the double-negative T_reg_ pool was comparable to GATA3^+^ T_regs_ (Fig. 2A). Beyond the colon, GATA3^+^ T_regs_ constitute 20 to 60% of the T_reg_ pools in various lymphoid and nonlymphoid tissues in adults. In contrast, RORγt^+^ T_regs_ remain largely restricted to the colon, where they represent 50% of the T_reg_ population (fig. S3A). We thus investigated whether the preferential expression of GATA3 by neonatal T_regs_ observed in the colon would be also present in other tissues. This trend was consistent across all tissues except the skin, where neonatal TagT_regs_ were significantly enriched for GATA3 expression compared to unTagT_regs_ (Fig. 2B). Conversely, RORγt expression was lower in TagT_regs_ across all tissues where it was detectable. We also identified a poorly described population of GATA3^+^RORγt^+^ T_regs_. They represented less than 10% of T_regs_ in the skin and colon and were almost undetectable in other tissues. As for RORγt^+^ T_regs_, their proportion was increased in unTag compared to TagT_regs_ (fig. S3B). These data indicate that neonatal T_regs_ preferentially adopt a GATA3^+^ fate, which persists into adulthood, shaping tissue specific T_reg_ phenotypes.

### Neonatal T_regs_ persisting in adults exhibit an activated phenotype

**Fig. 3.**
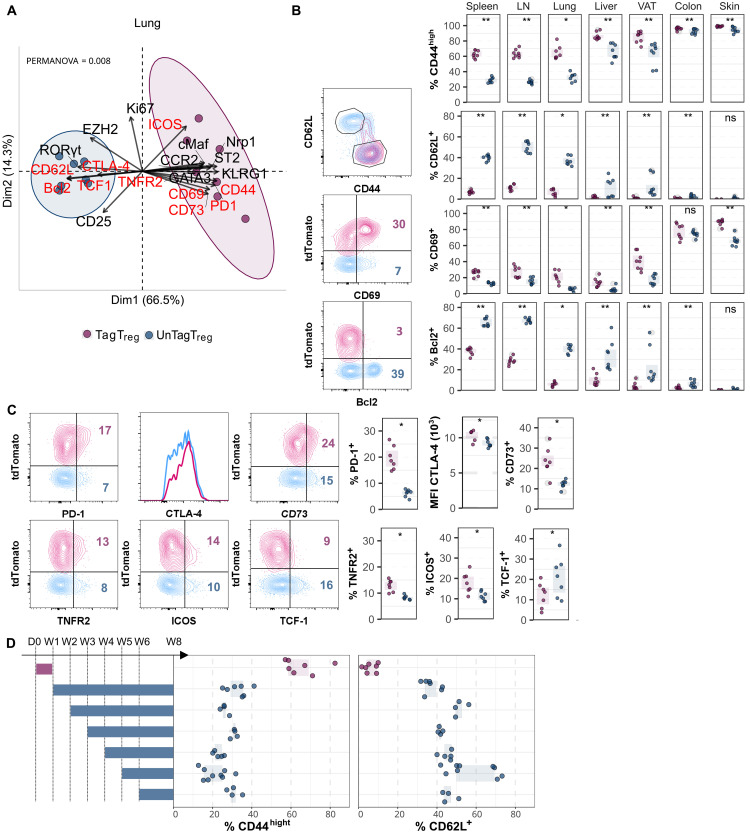
T_regs_ generated during the first week of life that persist into adulthood exhibit an activated phenotype. *Foxp3^iCre^ R26^Tom^* mice were treated with tamoxifen at week 1 (A to C) or at weeks 1 to 6 (D) and analyzed by flow cytometry at 8 weeks of age to compare the expression of activation markers in unTagT_regs_ (blue) and TagT_regs_ (purple). (**A**) PCA showing unTag and TagT_regs_ clusters in the lungs. Activation markers are in red. PERMANOVA, permutational multivariate analysis of variance. (**B** and **C**) Proportion of the indicated activation markers among unTag and TagT_regs_ in the seven analyzed tissues (B) and in the lungs (C). Representative density plots and histogram are shown. MFI, mean fluorescence intensity. (**D**) Proportions of CD44 and CD62L among TagT_regs_ in mice treated with tamoxifen at week 1 and among unTagT_regs_ in mice treated at weeks 1 to 6 as in Fig. 1 and analyzed in the lungs at 8 weeks of age. Experimental design is shown in the left. D0, day 0. Data were from four to eight mice per group pooled from two independent experiments. Statistical significance was calculated using a nonparametric Wilcoxon test. Each symbol represents one mouse, and the bars represent the medians. *P < 0.05 and **P < 0.01.

To further characterize neonatal T_regs_, we performed spectral flow cytometry using a 25-marker panel optimized for T_reg_ subset identification and functional profiling. Principal components analysis (PCA) revealed tissue-specific T_reg_ signatures, with skin and colon T_regs_ clustering separately due to differential expression of RORγt, CCR2, CD44, and CD69 (fig. S4A). We then treated *Foxp3^iCre^ R26^Tom^* mice with tamoxifen at week 1. For each of the seven analyzed tissues, unTag and TagT_regs_ clustered separately, exhibiting different phenotypes (fig. S4B). Neonatal TagT_regs_ exhibited an activated phenotype across all analyzed tissues. Specifically, in spleen, LNs, liver, and lungs, TagT_regs_ preferentially expressed CD44, CD69, CD73, CTLA4, ICOS, PD1, and TNFR2, whereas unTagT_regs_ were enriched for CD62L, Bcl2, and TCF1, markers associated with quiescence (Fig. 3, A to C, and fig. S5, A to C). A similar but less pronounced trend was observed in colon, VAT, and skin, where most T_regs_ exhibited an activated phenotype (Fig. 3B). We also compared CD44 and CD62L expression in TagT_regs_ generated at week 1 with unTagT_regs_ generated at weeks 2 to 6 in the lungs. Again, neonatal T_regs_ had a more activated phenotype than T_regs_ generated later on (Fig. 3D). Similar findings were obtained with Bcl2, PD1, and CTLA4 (fig. S5D). Our data show that neonatal T_regs_ that persist into adulthood exhibit an activated phenotype in various lymphoid and nonlymphoid tissues.

### Neonatal T_regs_ persisting in adults express tissue T_reg_ markers

**Fig. 4.**
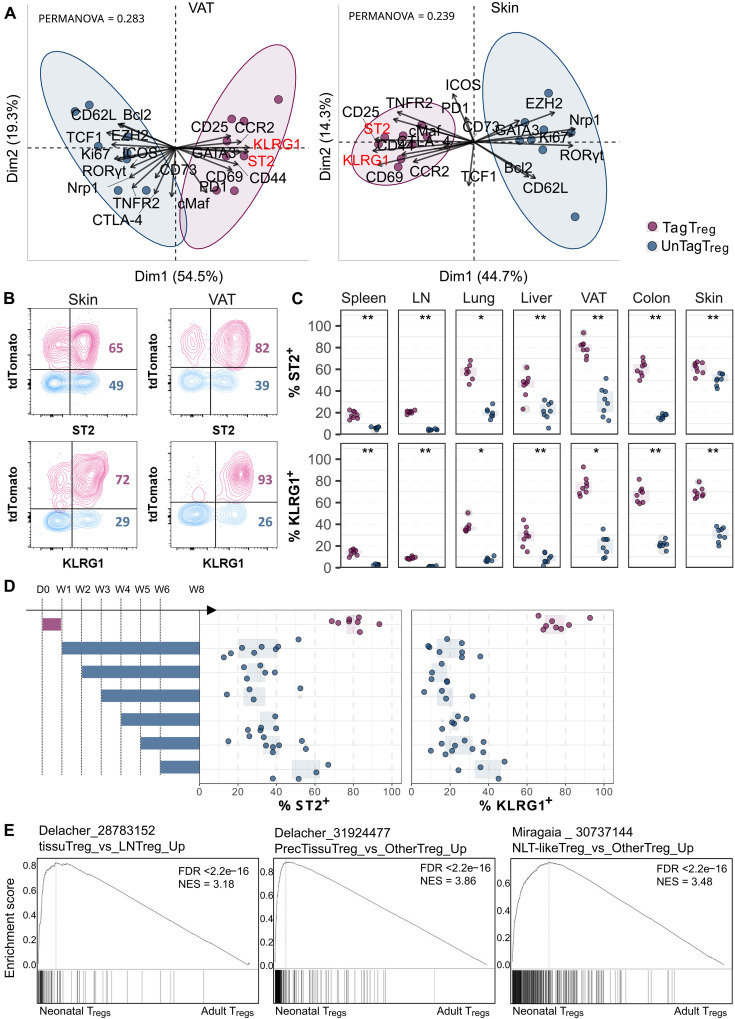
T_regs_ generated during the first week of life that persist into adulthood express tissue T_regs_ markers. *Foxp3^iCre^ R26^Tom^* mice were treated with tamoxifen at week 1 (A to C) or at weeks 1 to 6 (D) and analyzed by flow cytometry at 8 weeks of age to compare the expression of tissue T_reg_ markers in unTagT_regs_ (blue) and TagT_regs_ (purple). (**A**) PCA showing unTag and TagT_reg_ clusters in the VAT and skin. Tissue T_reg_ markers (KLRG1 and ST2) are colored in red. (**B**) Representative density plots showing ST2 versus KLRG1 expression among unTag and TagT_regs_ in the VAT and skin. (**C**) Proportion of cells expressing ST2 or KLRG1 among the unTag and TagT_regs_ in the seven analyzed tissues. (**D**) Proportions of ST2^+^ or KLRG1^+^ cells among TagT_regs_ in mice treated with tamoxifen at week 1 and among unTagT_regs_ in mice treated at different ages as in Fig. 1 and analyzed in the VAT at 8 weeks of age. Experimental design is shown in the left. Data were from four to eight mice per group pooled from two independent experiments. (**E**) Gene set enrichment analysis (GSEA) was performed to analyze whether the transcriptome of splenic neonatal T_regs_, compared to the one of adult T_regs_ (*9*), was enriched in a tissue T_reg_ signature (*14*) or a precursor T_reg_ signature (*15*, *17*). FDR, false discovery rate; NES, normalized enrichment score. Statistical significance was calculated using a nonparametric Wilcoxon test. Each symbol represents one mouse, and the bars represent the medians. *P < 0.05 and **P < 0.01.

We then assessed whether unTag and TagT_regs_ differently express markers of tissue T_regs_. KLRG1, ST2, and CCR2 are well-described tissue T_reg_ markers (*14*), and we confirm here their preferential expression in nonlymphoid tissue T_regs_ (fig. S3C). In lymphoid tissues, they have been described as markers of tissue T_reg_ precursors (*15*, *16*). In *Foxp3^iCre^ R26^Tom^* mice treated with tamoxifen at week 1, Tag and unTagT_regs_ of VAT and skin have distinct phenotype with preferential expression of KLRG1, ST2, and CCR2 in neonatal T_regs_ (Fig. 4A). In the VAT, colon, and skin, 60 to 80% of TagT_regs_ expressed these markers versus only 10 to 30% for unTagT_regs_ (except 50% for ST2 in the skin) (Fig. 4, B to D). Although attenuated, the same tendency was observed in other tissues. In the liver and lungs, 30 to 60% of TagT_regs_ expressed KLRG1 and ST2, values that dropped to 10 to 20% for unTagT_regs_. In the spleen and LNs, 10 to 20% of TagT_regs_ and less than 5% of unTagT_regs_ expressed these two markers (Fig. 4C). CCR2 expression was also higher in TagT_regs_ generated at week 1 compared to T_regs_ generated later on in most tissues (fig. S6). These data show that, in 8-week-old mice, neonatal T_regs_ expressed tissue T_reg_ markers at higher levels compared to T_regs_ generated later in life. To confirm these observations, we investigated whether neonatal T_regs_ were enriched for a tissue T_reg_ signature using published transcriptomic data. We analyzed differential transcriptomic data comparing neonatal and adult splenic T_regs_ (*9*), alongside a tissue T_reg_ signature established by comparing skin and VAT T_regs_ to LN T_regs_ in adult mice (*14*). This analysis revealed a strong enrichment of the tissue T_reg_ signature in neonatal T_regs_ compared to adult T_regs_ (Fig. 4E). Since tissue T_reg_ precursors have been identified in secondary lymphoid organs (*15*, *17*), we also examined whether neonatal T_regs_ expressed a signature enriched for these precursors. Again, we observed a strong enrichment of tissue T_reg_ precursor signatures in neonatal T_regs_ compared to adult T_regs_ (Fig. 4E). Thus, neonatal T_regs_ preferentially acquire tissue-resident characteristics and signatures and persist long-term in nonlymphoid compartments.

### T_reg_ turnover in aging mice

**Fig. 5.**
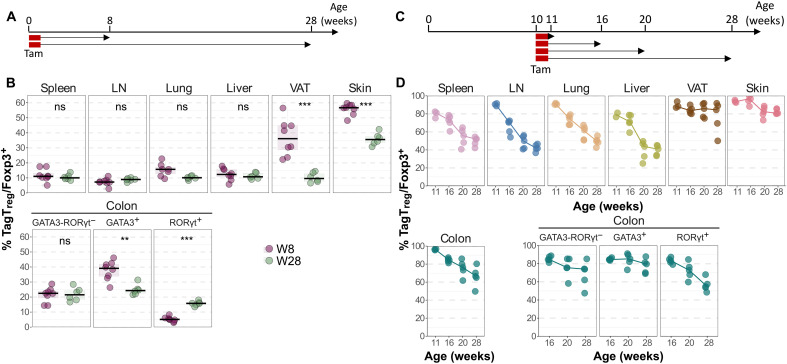
Persistence of neonatal T_regs_ in aged mice and T_reg_ renewal in adults. (**A** and **B**) *Foxp3^iCre^ R26^Tom^* mice were treated with tamoxifen at week 1 and analyzed at 8 or 28 weeks of age by flow cytometry. (A) Experimental design. (B) Proportion of TagT_regs_ among Foxp3^+^ cells in the spleen, LNs, lungs, liver, VAT, and skin (top) and in the GATA3^−^RORγt^−^, GATA3^+^, and RORγt^+^ T_reg_ populations of the colon (bottom). (**C** and **D**) *Foxp3^iCre^ R26^Tom^* mice were treated with tamoxifen at 10 weeks of age and analyzed at 11, 16, 20, and 28 weeks of age by flow cytometry. (C) Experimental design. (D) Proportions of TagT_regs_ among Foxp3^+^ cells in the spleen, LNs, lungs, liver, VAT, skin (top), and in the whole, GATA3^−^RORγt^−^, GATA3^+^, and RORγt^+^ T_reg_ populations of the colon (bottom). Data were from five to eight mice per group pooled from two independent experiments. Statistical significance was calculated using a nonparametric Wilcoxon test. Each symbol represents one mouse, and the bars represent the medians. **P < 0.01 and ***P < 0.001.

To assess whether neonatal T_regs_ persist beyond young adulthood, we examined the proportion of TagT_regs_ in 8-week-old and 28-week-old *Foxp3^iCre^ R26^Tom^* mice that had been labeled at week 1 (Fig. 5A). In spleen, LNs, lungs, and liver, neonatal T_regs_ constituted 10 to 15% of the T_reg_ pool at both ages. In contrast, in the skin, neonatal T_regs_ represented 60% at 8 weeks and still 35% at 28 weeks. In the VAT, neonatal T_regs_ dropped from 35 to 40% to 10% between 8 and 28 weeks (Fig. 5B). In the colon, the proportion of neonatal T_regs_ decreased among the GATA3^+^ T_reg_ subset and increased among the RORγt^+^ T_reg_ subset as the mice aged (Fig. 5B). To assess T_reg_ renewal in adults, we then treated *Foxp3^iCre^ R26^Tom^* mice with tamoxifen at 10 weeks and analyzed persistence of TagT_regs_ at 11, 16, 20, and 28 weeks (Fig. 5C). In lymphoid tissues, lungs, liver, and RORγt^+^ colonic T_regs_, we observed a gradual decrease of TagT_regs_ as the mice aged. In contrast, the proportion of TagT_regs_ stayed at a high level among VAT, skin, and GATA3^+^ colonic T_regs_ (Fig. 5D). These findings reinforce a model where some tissue T_reg_ niches are stably occupied by neonatal-derived cells, while others undergo continuous renewal throughout life.

### High proliferation of neonatal T_regs_ and residency of skin T_regs_

**Fig. 6.**
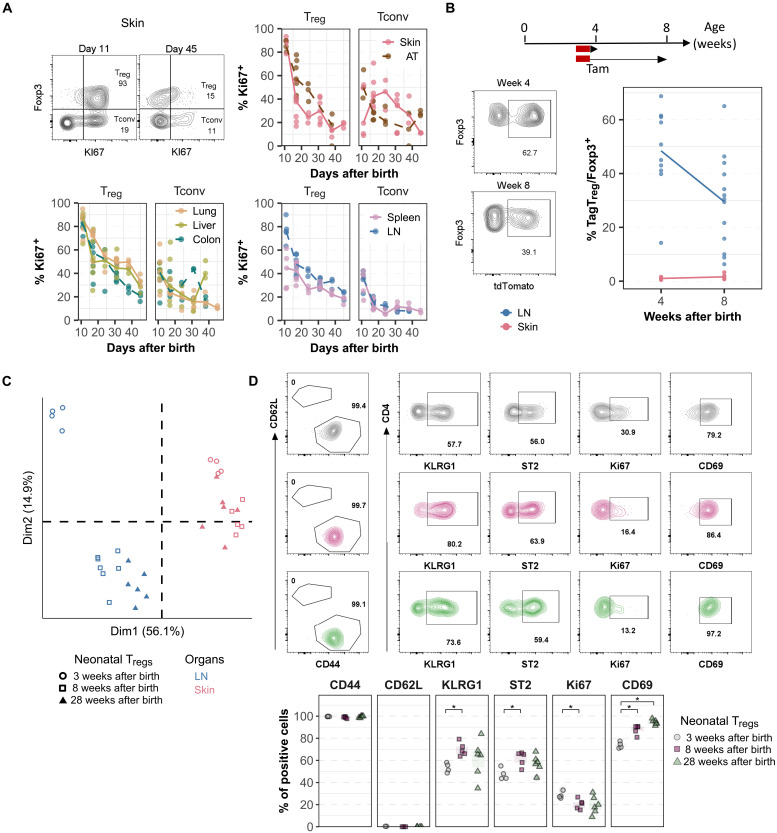
Proliferation and residency of neonatal skin T_regs_. (**A**) Proportion Ki67^+^ cells among T_regs_ or Tconvs in the spleen, LNs, lungs, liver, colon, adipose tissue (AT), and skin in 11- to 45-day-old mice. Top left: Representative density plots in the skin; shown values are the proportion of Ki67^+^ cells among T_reg_ or Tconv. (**B**) HO-Tam was applied locally on the skin of 3-week-old *Foxp3^iCre^ R26^Tom^* mice, and the proportion of TagT_regs_ was analyzed 1 or 5 weeks later. Experimental approach (top), representative density plots (bottom left), and proportion of TagT_regs_ among Foxp3^+^ cells in the skin and LNs (bottom right). (**C** and **D**) *Foxp3^iCre^ R26^Tom^* mice were treated with tamoxifen at 1 to 2 weeks of age, and the phenotype of TagT_regs_ was analyzed at 3, 8, or 28 weeks of age by flow cytometry. (C) PCA depicting the evolution of neonatal T_reg_ phenotype. (D) Representative density plots (top) and proportions (bottom) of CD62L, CD44, KLRG1, ST2, Ki67, and CD69 expression among neonatal T_regs_ analyzed at 3 (black), 8 (pink), or 28 (green) weeks of age. Data were from two (A and C) or three (B) independent experiments. Statistical significance was calculated using a nonparametric Wilcoxon test. Each symbol represents one mouse, and the bars represent the medians. *P < 0.05.

Our findings demonstrate that in certain tissues, such as the skin and VAT, T_regs_ generated during the neonatal period persist long-term and constitute a substantial fraction of the adult T_reg_ pool. To further investigate the biological properties of neonatal skin T_regs_, we analyzed their proliferation, colonization, and phenotypic evolution. While it is well known that T cells proliferate more during the neonatal period than in adulthood, the specific dynamics of T_regs_ compared to Tconvs remain less understood. To address this, we compared the proliferation of T_regs_ and Tconvs in lymphoid and nonlymphoid tissues during the first 2 months of life (Fig. 6A). In nonlymphoid tissues (including the skin, VAT, lungs, liver, and colon) as well as in LNs, the proportion of Ki67^+^ T_regs_ reached 80% or higher in most mice at 11 days of age. However, this proportion declined sharply in the skin, dropping to ~20% by 3 weeks of age (Fig. 6A). Other tissues exhibited similar declines, although at a more gradual pace, with Ki67^+^ T_regs_ reaching 20% by 6 to 8 weeks of age. In contrast, Tconvs proliferation followed a different trajectory: At 11 days of age, only 40% of Tconvs were Ki67^+^ across most tissues, and this proportion remained relatively stable or decreased only slightly in older mice. In the spleen, Ki67 expression was initially comparable between T_regs_ and Tconvs at 11 days of age, but after this stage, T_regs_ exhibited higher proliferation rates than Tconvs (Fig. 6A). To confirm the high proliferation rate of neonatal T_regs_ using a more direct technique, we measured incorporation of 5-ethynyl-2′-deoxyuridine (EdU) that was administered in vivo in 1-week-old- and 3-week-old mice. In both ages, T_regs_ proliferated substantially more than Tconvs in lymphoid and nonlymphoid tissues (fig. S7). T_reg_ proliferation was higher in 1-week-old mice compared to 3-week-old mice in the skin, confirming data obtained with the Ki67 staining. Collectively, these data reveal that neonatal T_regs_ proliferate notably more than Tconvs across all analyzed tissues before declining with age, particularly in the skin.

Our results reveal that by 8 weeks of age, 85% of the skin T_reg_ pool originates from T_regs_ generated before 2 to 3 weeks of age. However, whether these cells had already colonized the skin at the time of tamoxifen treatment remained unknown. Alternatively, TagT_regs_ may derive from precursors tagged in lymphoid tissues that later migrated to the skin or from T_regs_ tagged in other tissues that subsequently recirculated to the skin. To address this question, we applied 4-hydroxytamoxifen (HO-Tam) topically to the skin of 3-week-old *Foxp3^iCre^ R26^Tom^* mice (Fig. 6B). One week posttreatment, ~50% of skin T_regs_ were tagged, whereas T_regs_ in other tissues (draining LNs, spleen, and colon) remained untagged, confirming that labeling was restricted to the treated skin. To assess the long-term residency of these TagT_regs_, we quantified their proportion 5 weeks after tamoxifen application (Fig. 6B). In 8-week-old mice, TagT_regs_ still accounted for 30% of the total skin T_reg_ population, indicating that more than half of the skin T_regs_ present at 3 weeks persist into adulthood.

Last, we examined the phenotypic evolution of neonatal skin T_regs_ that persist in adulthood, comparing them to T_regs_ from 3-week-old mice. Using an optimized panel of 25 antibodies designed for T_reg_ subpopulation analysis, we found that neonatal skin T_regs_ that persist at 8 and even 28 weeks of age maintain a phenotype largely similar to that of 3-week-old skin T_regs_ (Fig. 6, C and D). In contrast, a parallel analysis of LN T_regs_ revealed a distinct pattern: Neonatal LN T_regs_ that persisted into adulthood exhibited substantial phenotypic changes compared to their 3-week-old counterparts (Fig. 6C and fig. S8). Collectively, these findings indicate that most of adult skin T_regs_ have already colonized the skin by 3 weeks of age and that their phenotype remains stable over time.

## DISCUSSION

Our study provides a comprehensive analysis of the kinetics of T_reg_ generation across various lymphoid and nonlymphoid tissues in adult mice. We observed major differences between tissues. In lymphoid organs, the T_reg_ pool is progressively constituted and continuously replenished, whereas in certain nonlymphoid tissues, such as the skin, most of the T_reg_ pool is established during the neonatal period and persists long-term.

In the spleen and LNs, our data show that ~10% of the adult T_reg_ pool is derived from cells generated during the first week of life, which are stably maintained for up to 28 weeks. The remaining population is progressively replaced by newly generated T_regs_ over time. The continuous renewal of T_regs_ in lymphoid organs is further supported by the gradual decline in TagT_regs_ observed in mice treated with tamoxifen at 10 weeks of age, suggesting ongoing replenishment as mice age.

The liver and lungs exhibit a distinct pattern of T_reg_ accumulation. While these tissues also undergo progressive replenishment, a higher fraction (40 to 50%) of the adult T_reg_ pool is generated before 2 weeks of age, compared to 25 to 35% in lymphoid organs. This early T_reg_ colonization may be linked to a peak in T_reg_ proportion between 2 and 3 weeks of age, as previously described (*4*, *6*, *15*), a phenomenon we confirm here. In the liver, early T_reg_ colonization is known to result from thymic output during the first week of life, followed by extensive in situ expansion to regulate Tconv activation (*6*).

The VAT exhibits an early T_reg_ generation, an intriguing finding given that this tissue is minimally developed in neonates. The location of the neonatal VAT T_reg_ pool remains uncertain, but we propose three nonmutually exclusive hypotheses: (i) T_regs_ may reside in lymphoid organs during early life before migrating to VAT, as previous studies have localized VAT T_reg_ precursors in the spleen and LNs of young mice (*15*, *16*); (ii) a small number of T_regs_ may initially colonize the nascent VAT, undergoing high in situ expansion as the tissue matures, a hypothesis compatible with our data showing a high rate of VAT T_reg_ proliferation in the first 3 weeks of life; (iii) neonatal T_regs_ may first reside in other adipose depots before later recirculating to the VAT. By 8 weeks of age, T_regs_ generated in the first week of life constitute 35% of the VAT T_reg_ pool, but this declines to 10% by 28 weeks. However, in 28-week-old mice treated with tamoxifen at 10 weeks, TagT_regs_ still account for 80% of VAT T_regs_, indicating a slow renewal between 10 and 28 weeks. A likely explanation of our results is that the pool of adult T_regs_ is mainly composed of cells generated during the first months of life. These findings align with reports that VAT T_regs_ in young and aged adults differ phenotypically, possibly due to population replacement over time (*18*).

The kinetics of T_reg_ generation in the skin is particularly unique. More than 55 and 85% of skin T_regs_ in 8-week-old mice originate from cells generated during the first week and first 2 weeks of life, respectively. Even at 28 weeks of age, T_regs_ generated in the first week of life still constitute 35% of the skin T_reg_ pool. The slow renewal of skin T_regs_ in adulthood is further confirmed by the sustained presence of TagT_regs_ in mice treated with tamoxifen at 10 weeks and analyzed 6 and 18 weeks later. Our findings are compatible with a previous study showing that skin T_reg_ precursors peak at 10 days of age in lymphoid tissues (*15*). Our findings are also consistent with the early and important colonization of the skin by activated T_regs_, which constitute up to 80% of CD4^+^ T cells in 2- to 3-week-old mice before declining to below 50% later in life [(*5*, *7*) and our data]. This early migration is driven by microbiota-induced CCL20 expression in hair follicles, which recruits CCR6^+^ T_regs_, highly enriched in the neonatal thymus (*8*). Neonatal skin T_regs_ are essential for inducing long-term immune tolerance to microbiota (*7*), a process that may be maintained by either the long-term persistence of neonatal T_regs_ or an infectious tolerance mechanism involving another regulatory cell player. Our data strongly support the former hypothesis, as most of skin T_regs_ in adulthood are of neonatal origin, which retain an activated phenotype and likely maintain strong suppressive function.

We also observed a systemic and high expansion of T_regs_ during the second week of life across all tissues except the spleen. This phenomenon was not observed in Tconvs. Neonatal T_reg_ expansion may be driven by lymphopenia-induced proliferation, a process previously described for T cells in neonates, but the differential proliferation of T_regs_ and Tconvs was not analyzed (*19*). T_regs_ have been described to undergo more robust expansion than Tconvs after adoptive transfer into lymphopenic adult mice (*20*), maybe explaining the greater expansion of T_regs_ over Tconvs in neonates. Prior studies have described high T_reg_ expansion in the neonatal liver (*4*, *6*). Two other studies have investigated T_reg_ proliferation in mice aged 2 weeks or more, i.e., after the stage of massive expansion that we reveal here. One showed slightly greater T_reg_ proliferation in skin than in lymphoid tissues (*5*), and the other slightly greater proliferation of T_regs_ than Tconvs in small intestine (*21*). Our study provides a longitudinal, multitissue analysis of this phenomenon. The systemic and massive expansion of neonatal T_regs_ may favor their tissue implantation and acquisition of their activated phenotype.

Another important finding is the phenotypic switch between neonatal-derived and later-generated T_regs_. Neonatal T_regs_ persisting into adulthood exhibit an activated phenotype, a phenomenon more visible in tissues with mixed populations of resting and activated T_regs_, such as the spleen, LNs, and lungs. This phenotype is less pronounced in the liver and VAT and difficult to observe in the colon and skin, where resting T_regs_ are rare. Similar observations have been described, but the reliability of the results was uncertain since only a fraction of neonatal T_regs_ was tagged, thus traced cells may not be representative of the entire population (*9*, *22*). The activated phenotype of neonatal T_regs_ persisting in adults may be driven by early lymphopenia-induced proliferation or their intrinsic higher self-reactivity compared to later-generated T_regs_ (*9*). To determine which of these two mechanisms is involved, one could compare the activation phenotype following adoptive transfer of either weakly self-reactive or strongly self-reactive T_regs_ into neonatal versus adult recipients. This activation state may be crucial for long-term immune regulation, particularly in controlling autoimmunity. Along these lines, it was shown that neonatal T_regs_ had a higher suppressive activity than adult T_regs_ in an in vitro assay and that neonatal T_regs_ that persist in adults were more efficient than adult T_regs_ to control an autoimmune syndrome after adoptive transfer in neonates (*9*).

In addition, we demonstrate that neonatal T_regs_ exhibit a type 2 immune profile, characterized by increased expression of GATA3 and ST2 compared to later-generated T_regs_, which could be related to the known type 2 immunity bias of the neonatal period (*1*). This finding may have important implications. VAT T_regs_, for example, are known to exhibit a T_H_2-like profile (GATA3^+^ST2^+^) (*23*), suggesting that their differentiation may be optimal in neonates because of its type 2 immune environment. This hypothesis is further supported by our data showing reduced GATA3 and ST2 expression in VAT T_regs_ generated after the neonatal period. A similar scenario may apply to skin T_regs_, which are predominantly generated in neonates and exhibit a T_H_2 profile (*24*). We observed a strong correlation between the proportion of GATA3^+^ T_regs_ and neonatal-derived T_regs_ across multiple tissues, reinforcing the hypothesis that the neonatal type 2 immune environment drives the differentiation of T_regs_ expressing markers of type 2 immunity (fig. S9).

The preferential differentiation of GATA3^+^ T_regs_ in neonates likely plays a crucial role in shaping the colon’s T_reg_ landscape. This tissue contains two well-characterized T_reg_ subsets, GATA3^+^ (also characterized by Helios expression) and RORγt^+^, which have distinct functions. Typically, these subsets have been attributed to thymic and peripheral origins, respectively, although this lineage segregation has recently been debated (*12*). Notably, studies have shown that in neonates, peripherally derived colonic T_regs_ express GATA3 and Helios but lack RORγt, a pattern not observed in older mice (*13*). As a result, the GATA3^+^/Helios^+^ subset predominates in the neonatal colon. Our findings support this, demonstrating that the neonatal period preferentially drives GATA3^+^ T_reg_ differentiation, affecting both thymic T_regs_ (across multiple tissues) and peripherally derived T_regs_ (specifically in the colon). Consistently, in the colon, we found that more than 60% of the GATA3^+^ T_reg_ pool in adult mice originates before 2 weeks of age, whereas only 10% of the RORγt^+^ T_reg_ pool is generated within this timeframe. These results suggest that the T_H_2 bias characteristic of neonates is critical for establishing the appropriate balance between these two T_reg_ subsets in the adult colon.

Our findings may have clinical relevance, particularly in the context of immunodysregulation polyendocrinopathy enteropathy X-linked (IPEX) syndrome, where T_reg_ deficiency results in severe T_H_2-skewed immunopathology (*25*), for which drugs neutralizing interleukin-4 (IL-4) and IL-13 are effective (*26*). In neonates, the preferential differentiation of GATA3^+^ T_regs_, known to better control type 2 immunity (*27*), may explain why patients with IPEX, who typically present in early childhood, exhibit type 2 immunopathology (*1*). Furthermore, our findings align with previous reports showing that neonatal T_reg_ depletion results in a T_H_2-biased immune response in adults (*11*, *28*), emphasizing the long-term impact of neonatal T_regs_ on immune homeostasis. Also, the capacity of skin T_regs_ to preferentially control local type 2 immunity has been reported (*24*, *29*), which is compatible with the long-term persistence of neonatal type 2 skin T_regs_ that we show here. All these experiments strongly suggest that neonatal T_regs_ persisting into adulthood preferentially regulate type 2 immunity. It would be difficult to further support this hypothesis using in vitro experiments because regulation of type 2 immunity by T_regs_ has been mostly documented in vivo.

Last, our skin-specific T_reg_ fate-mapping experiments confirm that skin T_regs_ exhibit high residency in young adults (3 to 8 weeks of age). A similar experiment performed in older adults indicate lower residency, with approximately a 50% decline in TagT_regs_ 3 weeks postlabeling (*30*). This suggests that skin T_reg_ residency is more pronounced in young adulthood, followed by increased replenishment. The transient residence of skin T_regs_ in adult mice was confirmed using parabiosis or specific markers (*31*–*33*). In the VAT, the residence of T_regs_ in adults would be of several weeks to months, as demonstrated in parabiosis and adoptive transfer experiments (*33*–*35*). Our findings challenge the recent concept of tissue-agnostic T_regs_, which posits that tissue-resident T_regs_ freely recirculate independently of their tissue of origin (*33*). However, T_reg_ recirculation from or to the skin or VAT was not analyzed in this report. Here, we show that T_regs_ resident in the skin and VAT are not tissue-agnostic since, if they were so, we would have the same proportion of TagT_regs_ between these tissues and the liver or the lungs, which is clearly not the case. Collectively, our experiments suggest a prolonged residence of tissue T_regs_ in young adults, at least in the skin. Then, the pool of skin and VAT T_regs_ is slowly replenished, either by T_reg_ recirculation between skin and VAT (plus possibly other unidentified tissues) or by precursors generated during the perinatal period.

In conclusion, our study provides a comprehensive analysis of the T_reg_ generation kinetics across multiple tissues. Our study underscores the critical role of the neonatal period in establishing long-lived T_reg_ populations specifically in the skin. These neonatal-derived T_regs_ exhibit an activated phenotype and a type 2 immune profile, suggesting specialized functions in long-term immune regulation. Understanding the ontogeny of T_regs_ provides key insights into immune tolerance and tissue-specific immune homeostasis in adulthood.

## MATERIALS AND METHODS

### Mice

*Foxp3^tm9(EGFP/cre/ERT2)Ayr^*/J (*Foxp3^iCre^*), B6.Cg-Gt(ROSA)26Sor^tm9(CAG-tdTomato)Hze/J^ (*R26^Tom^*), and *Foxp3^tm3(DTR/GFP)Ayr/J^* (*Foxp3^DTR^*) mice were purchased from the Jackson Laboratory. All mice were on a C57BL/6J ackground. Experiments were performed in both males and females, and since no differences were observed between sexes, the results were pooled and are presented together in the figures. They were housed under specific pathogen–free conditions. All experimental protocols were approved by our local Institutional Animal Care and Use Committees under the license numbers 02811.03, APAFIS#26677-202007211707430, and APAFIS#48261-2024031218097130. Mouse experimentation was performed in compliance with European Union guidelines.

### Tamoxifen and EdU administration

The tamoxifen (MP Biomedical, Ref: 11435161) was reconstituted at a final concentration of 40 mg/ml in peanut oil, obtained after 4 hours of vortex at 37°C. For mice older than 6 weeks, 8 mg of tamoxifen was administered orally by gavage. For mice younger than 6 weeks, tamoxifen was administered by two intraperitoneal injections, 3 days apart. They received a dose adapted to their age (0.1, 0.2, 1, and 2 mg for 1-, 2-, 3-, and 4- to 6-week-old mice, respectively). For the local treatment of the skin, HO-Tam (Sigma-Aldrich, Ref: T176) was resuspended at the final concentration of 20 μg/ml in acetone. Fifty microliters of the solution was applied directly on the ears during 4 consecutive days. EdU was administered intraperitoneally [at 5 mg/ml in phosphate-buffered saline (PBS)] three times on the day before tissue collection (at 7 a.m. and 1 and 7 p.m.). One-week-old mice received 50 μl per injection, whereas 3-week-old mice received 100 μl per injection. EdU incorporation was detected using the Click-iT Plus EdU Pacific Blue Flow Cytometry Assay Kit (Thermo Fisher Scientific, Ref: C10636), according to the manufacturer’s instructions.

### Preparation of cell suspensions

After cervical dislocation, mice were perfused intracardially with PBS to eliminate blood circulating cells. Lymphoid tissues (spleen and brachial/axillary LNs) and liver were collected in PBS complemented with 3% fetal calf serum (PBS-3%FCS). A single-cell suspension was obtained by mechanical dilaceration with a 70-μm cell strainer. The single-cell suspension obtained from the liver was resuspended in 40% Percoll, laid on an 80% Percoll, and centrifuged for 20 min at 2000 rpm at 20°C. Cells were collected at the 40%:80% Percoll interface and washed with PBS-3%FCS. The entire colon was collected, washed to remove feces, and cut longitudinally and then into small pieces. The tissues were subjected to three consecutive baths in order to remove the epithelium and collect the lamina propria: twice with PBS-3%FCS EDTA 5 mM and once with PBS-3%FCS, each lasting 20 min. After each bath, the tubes were vortexed for 10 s, and the pieces of tissue were transferred into the next tube. For the spleen and lungs, the red blood cells were lysed with ammonium chloride potassium for 1 min.

The VAT, lungs, and skin were collected in PBS and cut into small pieces before enzymatic digestion. For mice under 1 month old, the adipose tissue was collected subcutaneously. Digestion kits specific to each tissue from Miltenyi were used. The gentleMACS Dissociator was used for the lungs (Ref: 130-095-927) and the VAT (Ref: 130-105-808) in accordance with the instructions provided by the supplier. For the skin (Ref: 130-110-201) and the lamina propria (Ref: 130-097-410), the protocol was adapted, and the cells were digested using 12-well plates at 37°C with constant agitation for 105 and 30 min, respectively. After the digestion, the tissues were filtered with a 70-μm cell strainer. Enzymatic digestions had minimal impact on expression of surface molecules, as suggested from studying CD8a expression that is very sensitive to such treatment.

### Antibodies and flow cytometry

Cells were stained in 96-well plates with the antibodies and fluorescent reagent listed in Table 1. The viability of the cells was determined using a fixable live/dead dye (LIVE/DEAD Blue, Invitrogen). Nonspecific binding was limited by using the 2.4G2 hybridoma (anti-CD16/32). Membrane staining was performed for 20 min at 4°C in PBS-3%FCS. Fixation and permeabilization of the cells were performed using the Foxp3/Transcription Factor Staining Buffer Set from eBioscience (00-5523-00). Intracellular staining was performed for 45 min at 4°C using the buffer provided. Cells were acquired on the spectral cytometer Aurora from Cytek and analyzed using the FlowJo software. Cells treated with the Foxp3/Transcription Factor Staining Buffer from eBioscience maintained tdTomato expression. Combined with the use of the spectral cytometer Aurora from Cytek, we could clearly discriminate between tdTomato^+^ and tdTomato^−^ T_regs_.

**Table 1.** Antibodies used in flow cytometry. PE, phycoerythrin; Cy7, Cyanine 7; AP, allophycocyanin; PerCP, peridinin-chlorophyll-protein complex.

Target	Clone	Fluorochrome	Supplier	Reference
Bcl2	10C4	PE-Cy7	Invitrogen	25-6992-42
CCR2	475301	BUV661	BD Biosciences	750042
CD25	PC61	BV605	BioLegend	102036
CD3	145-2C11	BUV563	BD Biosciences	749277
CD4	GK1.5	BUV496	BD Biosciences	612952
CD44	IM7	PerCP	BioLegend	103035
CD45	30-F11	BUV395	BD Biosciences	564279
CD62L	MEL-14	BV650	BD Biosciences	564108
CD69	H1.2F3	BUV805	BD Biosciences	741927
CD73	TY/11.8	PerCP-Cy5.5	BioLegend	127214
CD8	53-6.7	PE-Cy5.5	Invitrogen	35-0081-80
c-Maf	sym0F1	eF450	Invitrogen	48-9855-42
CTLA-4	UC10-4F10-11	PE-CF594	BD Biosciences	564332
EZH2	11/EZH2	BV421	BD Biosciences	562963
Foxp3	FJK-16 s	FITC	Invitrogen	11-5773-82
GATA3	TWAJ	PE-Cy5	Invitrogen	15-9966-41
ICOS	7E.17G9	BV711	BD Biosciences	740763
Ki67	SolA15	AF532	Invitrogen	58-5698-82
KLRG1	2F1	BUV615	BD Biosciences	751191
NRP1	V46-1954	BV750	BD Biosciences	752452
PD-1	j43	BUV737	BD Biosciences	749422
RORγT	Q31-378	AF647	BD Biosciences	562682
ST2	U29-93	BV480	BD Biosciences	746701
TCF1	S33-966	R718	BD Biosciences	567587
TNFR2	REA228	APC	Miltenyi	130-123-275

### T_reg_ adoptive transfer in *Foxp3^DTR^* mice

Single-cell suspension obtained from the spleen and LNs of the *Foxp3^iCre^ R26^Tom^* donor mice were stained with an anti-CD25 (7D4) biotin–labeled antibody and were then coated with anti-biotin microbead (Miltenyi, Ref: 130-090-485). After magnetic sorting, cells of the CD25^+^ enriched positive fraction were labeled with the carboxyfluorescein diacetate succinimidyl ester (CFSE) CellTrace kit from eBioscience and were intravenously injected into *Foxp3^DTR^* recipient mice (10^6^ cells per mouse). The recipient mice were treated with tamoxifen (two times, 2 mg) and diphtheria toxin (three times, 1 μg) by intraperitoneal injections before the adoptive transfer. The donor cells were identified by flow cytometry in the spleen, LNs, lungs, liver, and colon based on the CFSE expression 5 days after the adoptive transfer.

### Flow cytometry analysis, GSEA, biostatistic, and data modelization

Raw FCS data from the Aurora were analyzed using FlowJo software to produce data tables. The following analysis was performed using R (4.3.1). PCA was performed using factominer (2.8) and factoextra (1.0.7). Boxplots and line plots were generated using ggplot2 (3.4.3). Statistical analyses were performed using rstatix (0.7.2) and ggpubr (0.6.0). Gene set enrichment analysis (GSEA) was performed using the Webgestalt software (https://www.webgestalt.org/). Normalized gene expression data were used as input, along with a preranked list of genes based on their correlation with the experimental condition [log_2_ fold change between neonatal and adult T_regs_ obtained from Yang *et al.* (*9*)]. The analysis was conducted in preranked mode with 1000 permutations, using the gene set of VAT- and skin T_regs_ versus LN T_regs_ (*14*) or the gene sets of tissue T_reg_ precursors versus other T_regs_ in the spleen, identified by their Nfilt3^+^Klrg1^+^ or nonlymphoid like T_reg_ phenotype (*15*, *17*).
