# Supplementary material for: Neonatal regulatory T cells persist into adulthood across multiple tissues with high enrichment in the skin
Source: Sci Adv. 2025 Oct 3;11(40):eadx8037. doi: 10.1126/sciadv.adx8037 (PMC12494032; doi:10.1126/sciadv.adx8037)
Supplement: Supplementary file 1 — Figs. S1 to S9 [file sciadv.adx8037_sm.pdf]

Science Advances
AAAS

Supplementary Materials for

Neonatal regulatory T cells persist into adulthood across multiple tissues with high enrichment in the skin

Morgane Hilaire *et al.*

Corresponding author: Benoît L. Salomon, benoit.salomon@inserm.fr;
Morgane Hilaire, hilaire.morgane77@gmail.com

Sci. Adv. **11**, eadx8037 (2025)
DOI: 10.1126/sciadv.adx8037

This PDF file includes:

Figs. S1 to S9

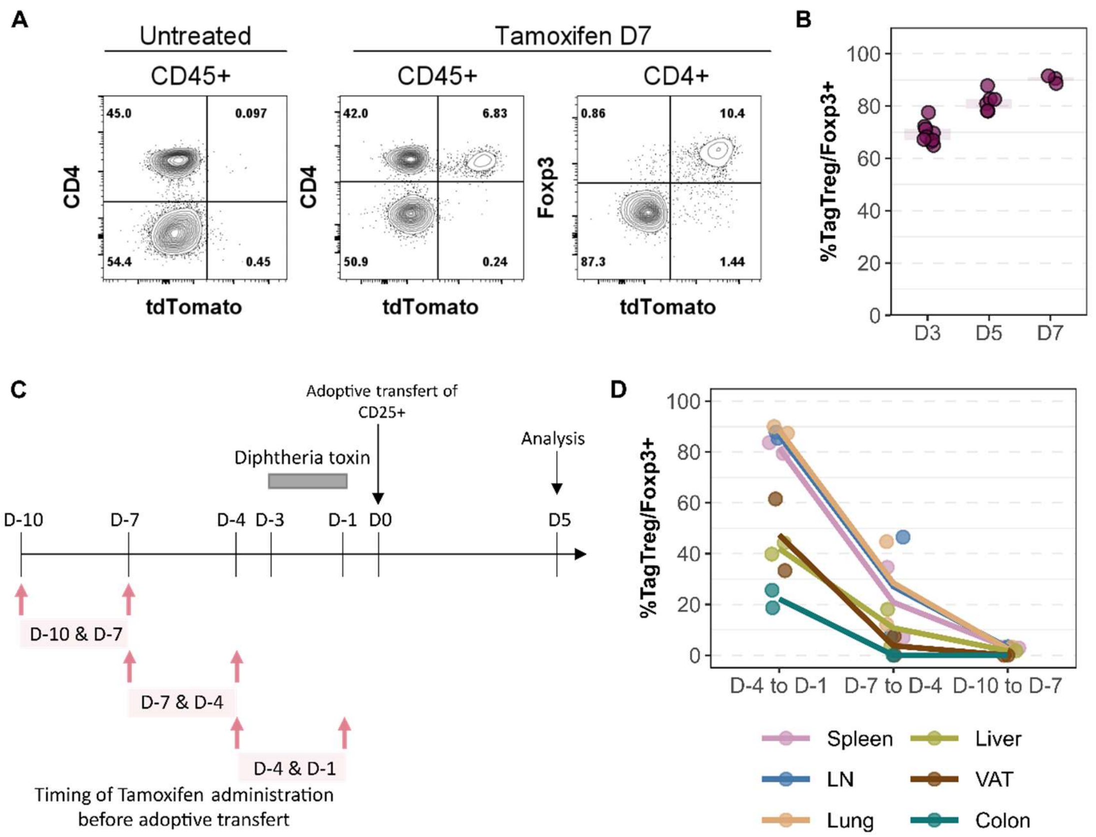

Fig. S1. Efficiency and duration of the tamoxifen effect. (*A*) Representative dot plot of tdTomato vs CD4 or Foxp3 in CD45$^+$ or CD4$^+$ splenocytes of *Foxp3^{iCre} R26^{Tom}* mice untreated or 7-days post tamoxifen treatment. (*B*) Proportion of TagTregs among Foxp3$^+$ cells in *Foxp3^{iCre} R26^{Tom}* mice 3, 5 and 7 days after tamoxifen treatment. (*C*, *D*) *Foxp3^{DTR}* recipient mice received 2 injections of tamoxifen at different times prior to the adoptive transfer of purified CD25$^+$ cells from *Foxp3^{iCre} R26^{Tom}* donor mice. Recipient mice were treated with diphtheria toxin before the adoptive cell transfer. (*C*) Experimental design. (*D*) Proportions of TagTregs among donor Foxp3$^+$ cells, determined 5 days after adoptive transfer in the spleen, LNs, lungs, liver, VAT and colon.

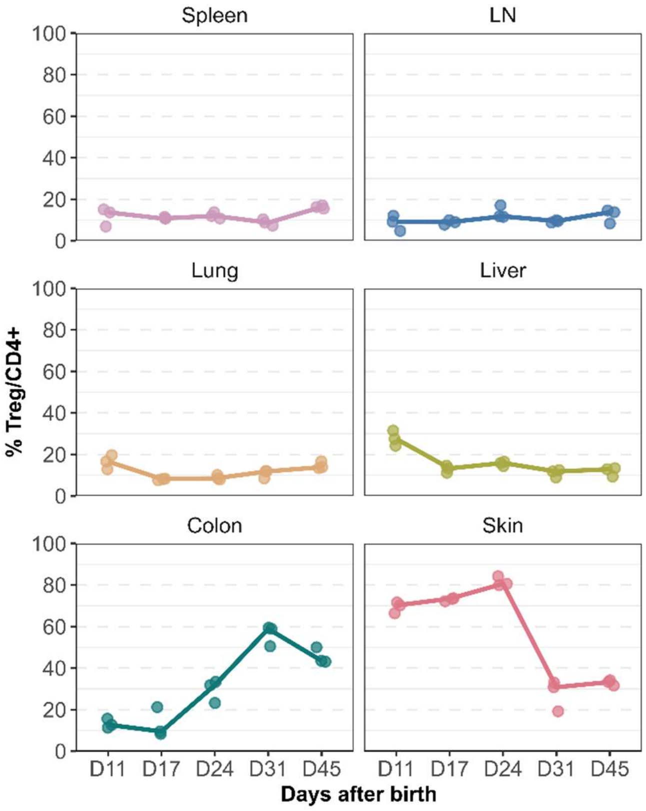

Fig. S2. Treg proportions in indicated tissues during the first 45 days of life. Treg proportion among $CD4^+$ T cells determined in the spleen, LNs, lungs, liver, colon and skin in 11 to 45 days old mice. Each symbol represents one mouse. One of 2 representative experiments is shown.

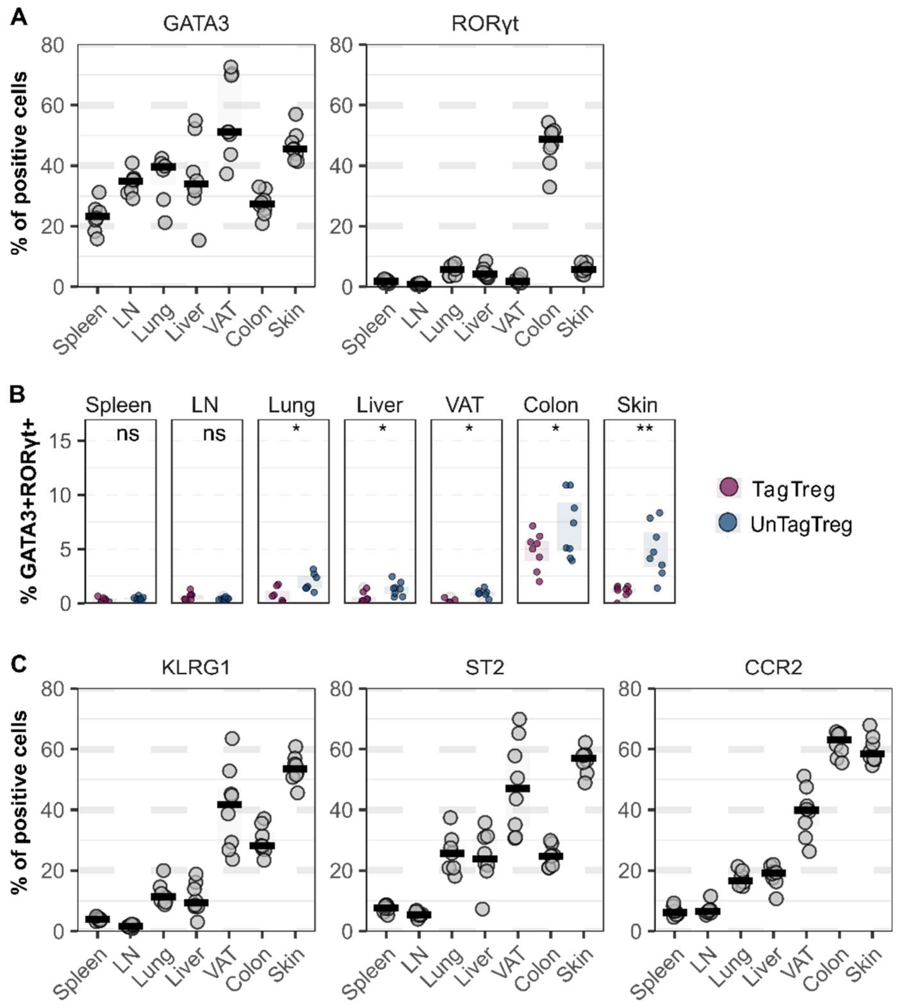

Fig. S3. Expression of different Treg markers in 8-week-old mice. (*A*) Proportion of Tregs expressing GATA3 and RORγt in the spleen, LNs, lungs, liver, VAT, colon, and skin. (*B*) *Foxp3*^{iCre} *R26*^{Tom} mice were treated with tamoxifen at week 1 and analyzed at 8 weeks of age to compare the proportion of GATA3^{+}RORγt^{+} among unTag and TagTregs in the 7 analyzed tissues. (*C*) Proportion of Tregs expressing KLRG1, ST2, and CCR2 in the spleen, LNs, lungs, liver, VAT, colon, and skin. Each symbol represents one mouse, and the bars represent the medians from at least 2 independent experiments.

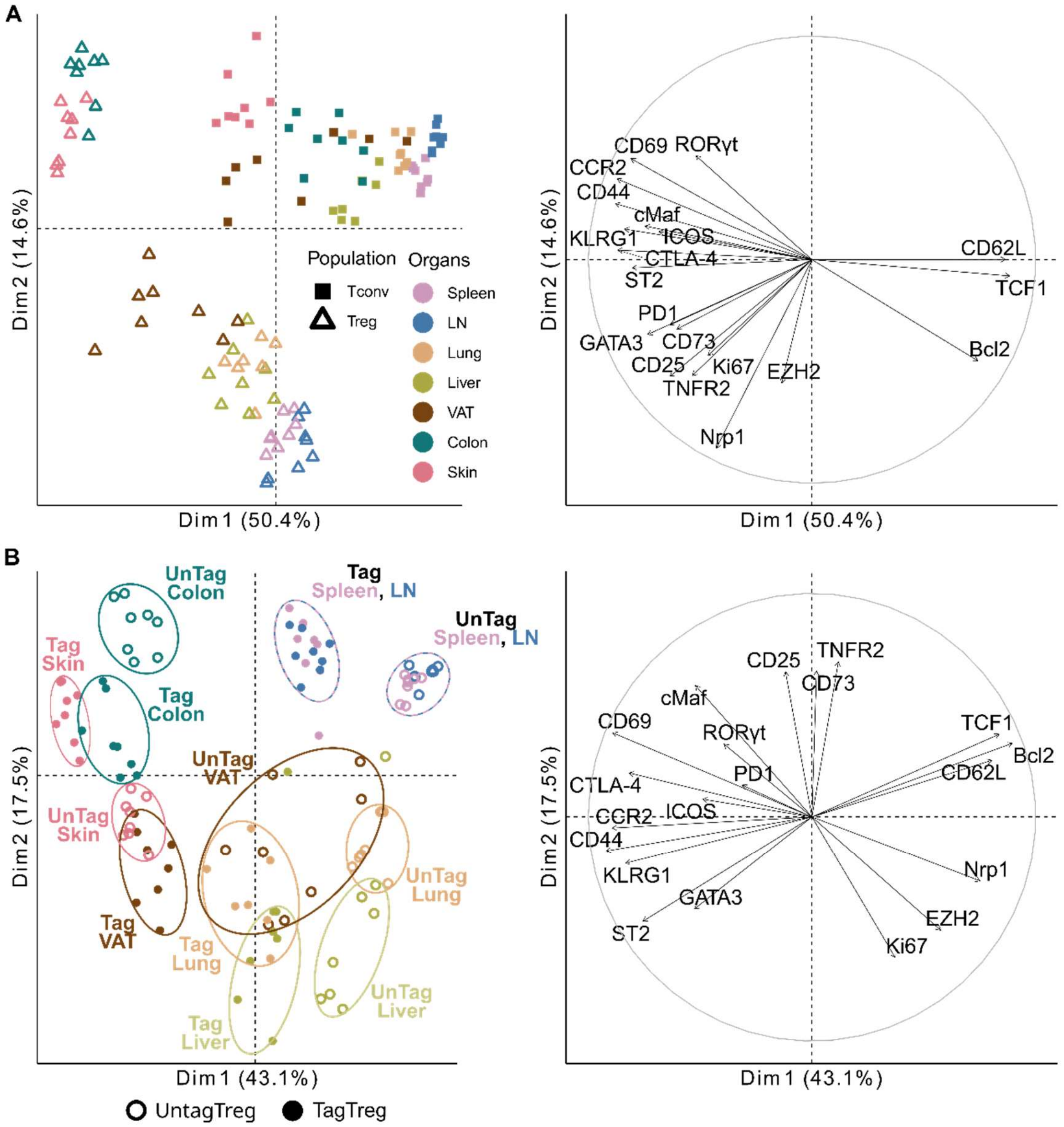

Fig. S4. Phenotypic discrimination of Tconvs, unTagTregs and TagTregs from different tissues. Cells of 7 tissues (spleen, LNs, liver, lungs, colon, skin and VAT) were analyzed by flow cytometry using a mixed panel of monoclonal antibodies designed to characterize Treg subsets and activation (see methods). *Foxp3^{iCre} R26^{Tom}* mice were treated with tamoxifen at week 1 and analyzed at 8 weeks of age. PCA showing the clusters of Tconvs and all Tregs (*A*) and the clusters of unTagTregs and TagTregs (*B*) in the different tissues. For the 20 markers that drive the PCA, the direction and size of each arrow show the contribution of each marker to the variance of the PCA (right panels). Ellipses encircle the unTag and TagTregs of each organ. Data were from 2 independent experiments.

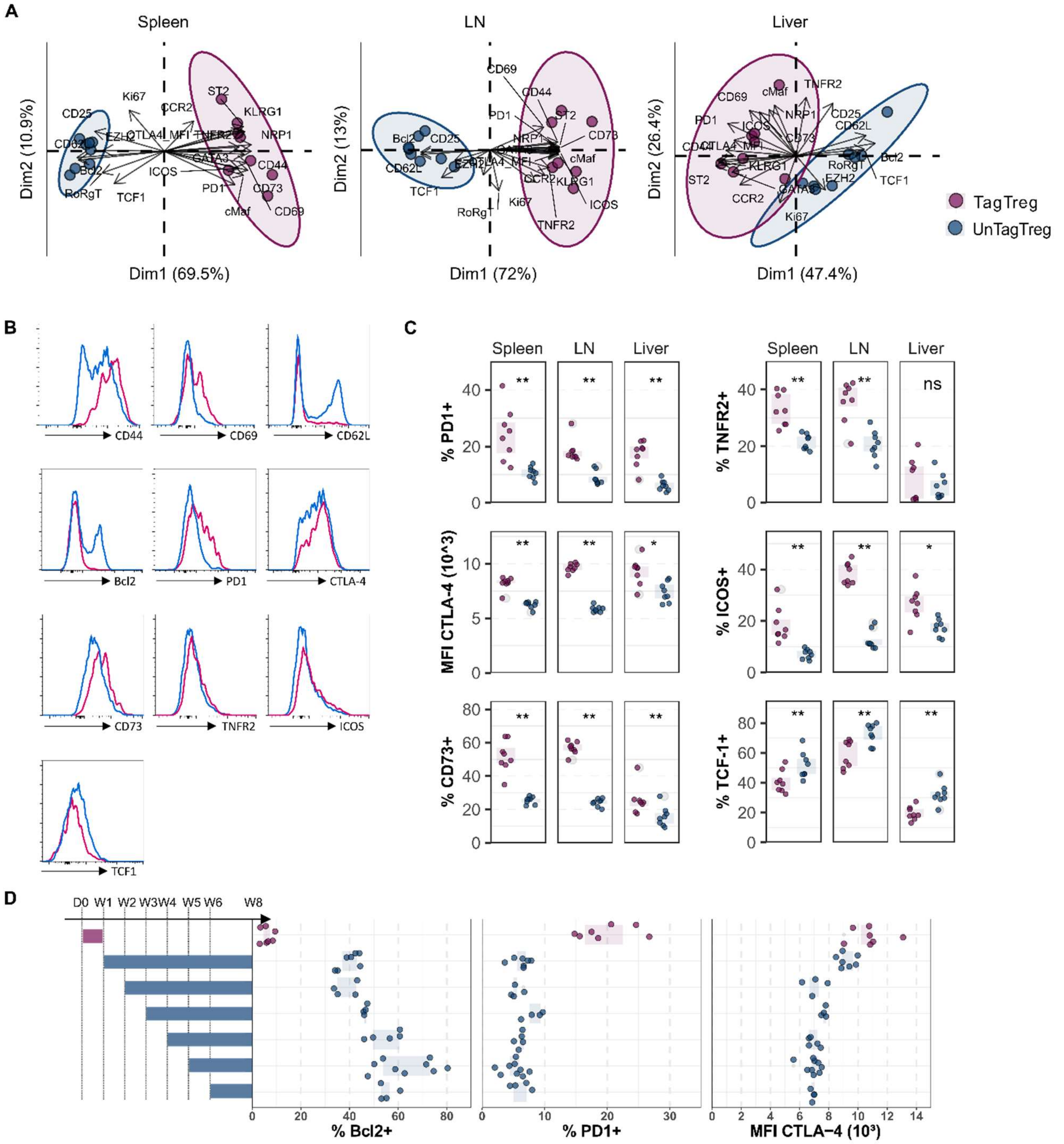

Fig. S5. Expression of activation markers among neonatal TagTregs that persist into adulthood. *Foxp3^{iCre} R26^{Tom}* mice were treated with tamoxifen at week 1 (*A-C*) or at week 1 to 6 (*D*) and analyzed by flow cytometry at 8 weeks of age to compare the expression of activation markers in unTagTregs (blue) and TagTregs (purple). (*A*) PCA showing the unTag and TagTreg clusters in the spleen, LNs and liver. Representative histograms in the lungs (*B*) and proportion (*C*) of the indicated activation markers among the unTag and TagTregs in the spleen, LNs and liver. (*D*) Proportions of Bcl2^{+}, PD1^{+} cells and MFI of CTLA4 among TagTregs in mice treated with tamoxifen at week 1 and among unTagTregs in mice treated at week 1 to 6 as in figure 1 and analyzed in the lungs at 8 weeks of age. Experimental design is shown in the left panel. Data were from 4-8 mice per group pooled from 2

independent experiments. Statistical significance was calculated using a non-parametric Wilcoxon test. Each symbol represents one mouse and the bars represent the medians. *P < 0.05, **P < 0.01.

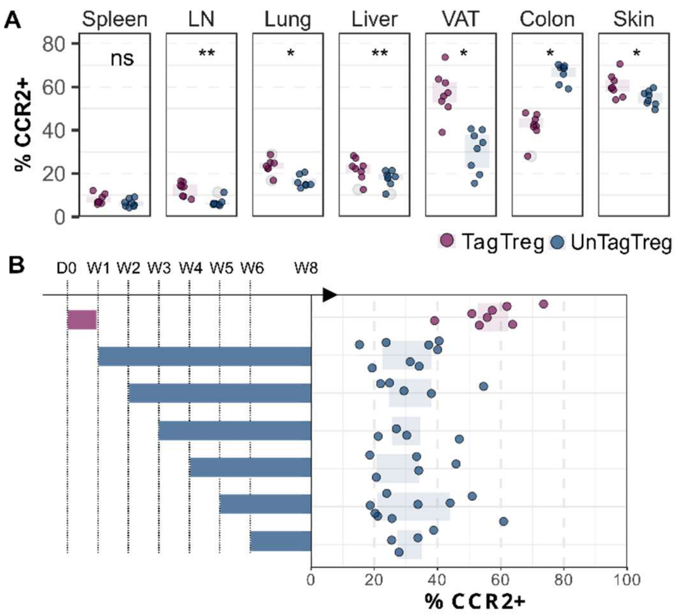

Fig. S6. Expression of CCR2 in unTagTregs (blue) and TagTregs (purple). *Foxp3*^{iCre} *R26*^{Tom} mice were treated with tamoxifen at week 1 (*A*) or at week 1 to 6 as in figure 1 (*B*) and analyzed by flow cytometry at 8 weeks of age. (*A*) Proportion of CCR2 among unTag and TagTregs in the spleen, LNs, lungs, liver, VAT, Colon and skin. (*B*) Proportions of CCR2 among TagTregs in mice treated with tamoxifen at week 1 and among unTagTregs in mice treated at week 1 to 6 as in figure 1 and analyzed in the VAT. Experimental design is shown in the left panel. Data were from 4-8 mice per group pooled from 2 independent experiments. Statistical significance was calculated using a non-parametric Wilcoxon test. Each symbol represents one mouse and the bars represent the medians. *P < 0.05, **P < 0.01.

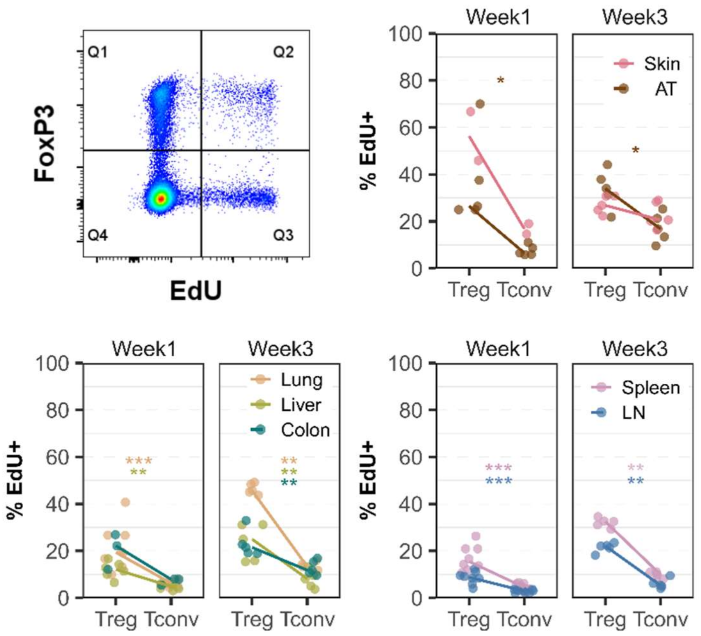

Fig. S7. Incorporation of EdU in vivo in one week-old- and three-week-old mice. EdU was administered in one week-old- and three-week-old mice three times on the day prior to tissue collection. Proportion of EdU$^+$ among Tregs and Tconvs in the spleen, LNs, lungs, liver, AT, Colon and skin. A representative dot plot is shown for the LNs. Statistical significance was calculated using a non-parametric Wilcoxon test. Each symbol represents one mouse and the lines depict connections between medians. *P < 0.05, **P < 0.01, ***P < 0.001.

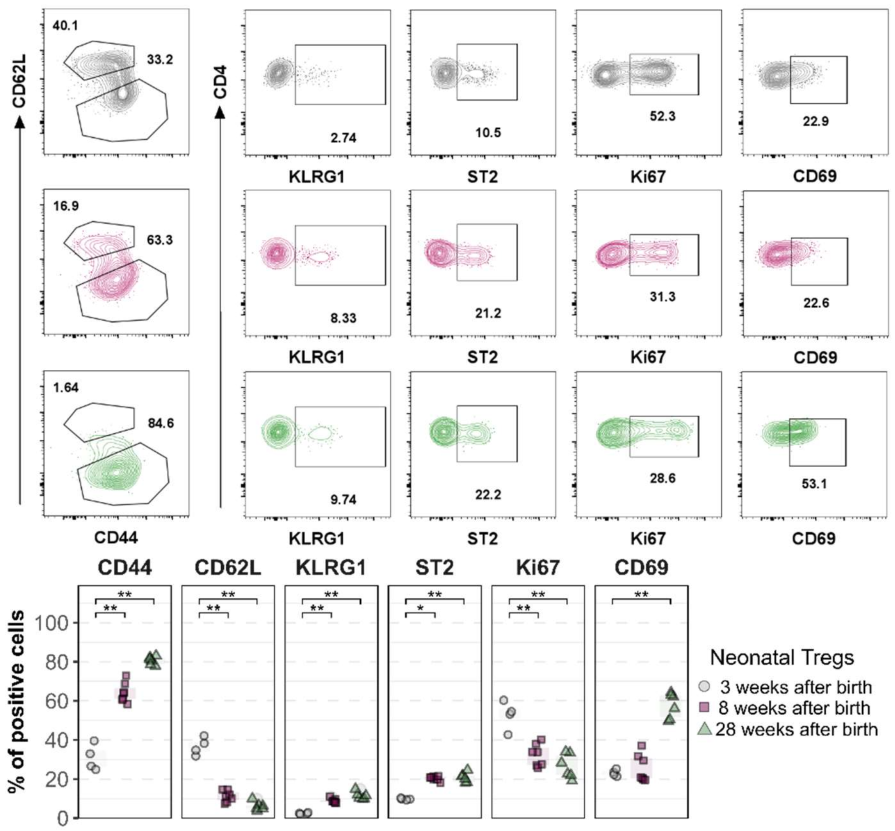

Fig. S8. Phenotype of neonatal Tregs persisting in adulthood in the LNs. *Foxp3*^{iCre} *R26*^{Tom} mice were treated with tamoxifen at 1-2 weeks of age and the phenotype of TagTregs were analyzed at 3 (black), 8 (pink) or 28 (green) weeks of age by flow cytometry. Representative density plots (upper panels) and proportions (lower panels) of CD62L, CD44, KLRG1, ST2, Ki67 and CD69 expression among neonatal Tregs. Data were from 2 independent experiments. Statistical significance was calculated using a non-parametric Wilcoxon test. Each symbol represents one mouse and the bars represent the medians. *P < 0.05, **P < 0.01.

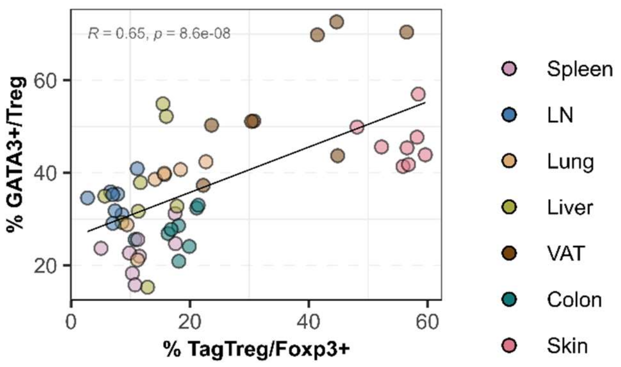

Fig. S9. Correlation between GATA3⁺ Tregs and proportion of TagTreg. *Foxp3^{iCre} R26^{Tom}* mice were treated with tamoxifen at week 1 and analyzed at 8 weeks of age by flow cytometry. Correlation between the proportion TagTregs and GATA3^{+} among Foxp3^{+} cells in the indicated tissues. Each symbol represents one mouse. Statistical significance was determined using the Pearson correlation coefficient (R). Data were from 2 independent experiments.
